# Assessment of Implementation of Integrated Management of Neonatal and Childhood Illness in India

**DOI:** 10.3329/jhpn.v29i6.9900

**Published:** 2011-12

**Authors:** Pavitra Mohan, Baya Kishore, Sharad Singh, Rajiv Bahl, Anju Puri, Rajesh Kumar

**Affiliations:** ^1^UNICEF India Country Office, New Delhi, India; ^2^Ministry of Health and Family Welfare, Government of India, New Delhi, India; ^3^School of Public Health, Postgraduate Institute of Medical Education and Research, Chandigarh, India; ^4^Department of Child and Adolescent Health, World Health Organization, Geneva, Switzerland

**Keywords:** Child survival, Infant health, Integrated management of childhood illness, Neonatal mortality, Newborn care, Performance evaluation, India

## Abstract

At the current rate of decline in infant mortality, India is unlikely to achieve the Millennium Development Goal on child survival. Integrated Management of Neonatal and Childhood Illness (IMNCI), adapted from the global Integrated Management of Childhood Illness to enhance the focus on newborns and on community health workers, is the central strategy within the National Reproductive and Child Health Programme to address high infant mortality. This paper assessed the progress of IMNCI in India, identified the programme bottlenecks, and also assessed the effect on coverage of key newborn and childcare practices. Programme data were analyzed to ascertain the implementation status; rapid programme assessment was conducted for identifying the programme bottlenecks; and results of analysis of two rounds of district-level household surveys were used for comparing the change in the coverage of child-health interventions in IMNCI and control districts. More than 200,000 community health workers and first-level healthcare providers were trained during 2005-2009 at a variable pace across 223 districts. Of the reported births (n=1,102,573), 65.5% were visited by a trained worker within 24 hours, and 63.1% were visited three times within 10 days. Poor supervision and inadequate essential supplies affected the performance of trained workers. During 2004-2008, 12 early-implementing districts had covered most key newborn and child practice indicators compared to the control districts; however, the difference was significant only for care-seeking for acute respiratory infection (net difference: 17.8%; 95% confidence interval 2.3-33.2, p<0.026). Based on the early experience of IMNCI implementation in different states of India, measures need to be taken to improve supportive supervision, availability of essential supplies, and monitoring of the programme if the strategy has to translate into improved child survival in India.

## INTRODUCTION

The pace of decline in the infant mortality rate (IMR) in India is insufficient to achieve the targets set up under the XI National Five Year Plan, or to reach the Millennium Development Goals (1). The main causes of infant deaths remain perinatal conditions (46%), respiratory infections (22%), and diarrhoea (10%) (2).

One of the major reasons for the slow decline in the IMR is the stagnation in neonatal mortality. In the current decade, neonatal mortality is declining sluggishly, moving from 40 per 1,000 livebirths in 2001 to 36 per 1,000 livebirths in 2007. Within the neonatal period, 80% of all newborn deaths occur during the first week of life—the early neonatal period (3). The major causes of neonatal mortality are severe infections (36%), birth asphyxia (23%), and preterm births (25%) (4).

The coverage of child-health interventions remains highly inadequate in India. The third National Family Health Survey (NFHS III) data showed that, in 2005-2006, only 43.5% of children were fully immunized; 26.2% of children, aged less than three years (under-3 children), suffering from diarrhoea, received oral rehydration salt (ORS) solution; and 64.2% of children with acute respiratory infection (ARI) or fever in the last two weeks were taken to a health facility. Only 23% of under-three children received breastfeeding within one hour of birth, and 46.3% aged 0-5 months were exclusively breastfed (5).

The Reproductive and Child Health Programme Phase II (RCH II) was launched in India in 2005. The programme planned a comprehensive package of newborn and child-health interventions aiming at achieving a decisive decline in neonatal, infant and child mortality. Integrated Management of Neonatal and Childhood Illness (IMNCI), adapted from the global Integrated Management of Childhood Illness (IMCI) package, formed the central strategy for child survival, with skilled attendance at birth for all newborns and immunization-plus being the other two pillars. The aim was to implement IMNCI at the household level in 125 districts and at the facility level across the country by 2010 (6).

The IMCI strategy was adapted to IMNCI in India, based on the recognition that, globally, a limited number of childhood illnesses, such as pneumonia, diarrhoea, measles, malaria, and malnutrition, kill 70% of under-five children. The guidelines relied on the detection of cases using simple clinical signs without laboratory tests and offered empirical treatment. IMCI only covered children aged seven days to five years (excluding the early neonatal period) and targeted health workers at primary-care facilities (7).

Two features distinguish this approach from the generic IMCI. Recognizing newborn care as critical for improving child survival, it was strengthened in IMNCI by increasing the newborn-care component of the training programme and including prevention and management of health conditions in the first week of life. Second, recognizing that a large proportion of sick children do not come in contact with health workers but most of them can be reached by community-based workers, IMNCI in India focuses on community-based rather than facility-based healthcare providers. To ensure focus on newborns, home-visits to all newborns in the first week of life by the community-based workers (Anganwadi workers−AWWs and/or accredited social health activists−ASHAs) for the promotion of optimal care practices and identification of severe illness and referral, have been included as a key operational element (8,9). The AWWs manage a village-level community nutrition centre, called Anganwadi, and provide a set of services for promoting the growth and development of under-six children. They receive a fixed remuneration for the services. The ASHAs are community health ‘activists’ whose primary role is to associate families with health services and are provided performance-based incentives for this role.

### Description of intervention

#### Training

The IMNCI training programme focuses on building of individual skills and includes practice sessions in the field and in the hospital. Each training programme is run for eight days. The training batch is restricted to about 24 participants with the facilitator-participant ratio of about 1:5. Frontline community-based workers and auxiliary nursemidwives (ANMs) are trained together in basic health workers course. Supervisors are trained additionally in a two-day course (10-12).

#### Provision of care by IMNCI-trained workers

Following training, workers are supposed to make home-visits to all newborns within their areas on day 1, 3, and 7 of life. During these visits, the health workers assess the newborns, ensure breastfeeding, counsel on warmth and danger-signs, treat local infections, and refer to appropriate facilities for possible serious bacterial infections. In addition, the workers are expected to assess sick children, manage children with minor illness, and refer severelyill children. The workers are supposed to receive basic drugs and supplies as per the guidelines of the RCH II and Integrated Child Development Scheme (ICDS) programmes. Line supervisors are supposed to supervise the trained workers, using the structured supervisory checklists.

### Purpose of this paper

IMNCI has been implemented in India for about five years. There is limited information and evidence on the quality of its implementation, operational constraints, and facilitating factors and its effectiveness in improving the coverage of key newborn and childcare practices and interventions. Such information is critical to guide the further implementation of the programme and to modify the course. This information will also be useful to other countries that are implementing large-scale community-based newborn and child-health interventions. This paper describes the scaling-up of IMNCI in India and summarizes the current status of its implementation. While doing so, it seeks to answer the following questions:

- What has been the pace of training a large number of health workers (HWs) and community health workers (CHWs) on skill-based training using the IMNCI approach?
- What was the quality of training when the programme was scaled up?
- Following the training, how did the HWs and CHWs perform? What proportion of newborns was the CHW able to visit in the first week of life? What was the quality of care they provided to sick infants and children?
- What programme bottlenecks affect the effective implementation of the strategy and how have they been addressed so far, if at all?
- What has been the effectiveness of IMNCI, if any, on the coverage of some key newborn and child-health practices and services in the districts implementing IMNCI?

## MATERIALS AND METHODS

The paper is based on review of information generated from different sources, using a mix of methods as described below.

### Methods for assessing coverage, pace, and quality of training

The districts implementing IMNCI routinely report on the progress of IMNCI training and implementation. Reports from each of these districts were collated, with assistance of the state offices of the United Nations Children's Fund (UNICEF). Data were triangulated with the data reported in the routine Health Management Information System (HMIS) for the National Rural Health Mission (NRHM) by the states to the Government of India. Incomplete and inconsistent data were corrected by contacting and seeking clarifications from the district and state authorities.

Based on the progress of IMNCI implementation, districts were categorized into: (a) introductory phase (0-3 months after inclusion in the state's Project Implementation Plan), (b) early-implementation phase (up to 50% training load completed), (c) expansion phase (50-90% training load completed), and (d) consolidation phase (more than 90% training load completed). Of the 627 districts in India, 223 (35.56%) initiated the IMNCI programme during 2005-2009. A detailed report on progress in training was available for 99 (44.39) of the 223 districts. Table 1 shows the indicators included to describe the progress of IMNCI training.

The quality of training programmes was also assessed based on quality checks on a random subset of 70 training programmes across 14 states. The trained supervisors conducted the quality checks using a standard tool. The tool scores on different dimensions of quality, and the range of score achievable is 0-100. The median quality score was used as the indicator for the quality of training programme.

### Performance of trained health workers and community health workers

#### Coverage of home-visits

After training, the community health workers recorded the home-visits in a case record form. They then reported on the key coverage indicators. The reports were progressively collated at the sub-centre, Primary Health Centre (PHC), and at district level. The performance of the health workers on the coverage of home-visits to newborns was assessed from the monthly reports. The key indicators used for assessing the coverage of home-visits are shown in Table 1.

#### Quality of home-visits

In three states, the trained supervisors assessed the performance of a sample of community workers during home-visits to newborns using a standard checklist. The proportions of workers who appropriately classified, identified treatment, and counselled were used as key indicators to assess the quality of home-visits.

Besides, their performance was also assessed during the rapid programme assessment of IMNCI in seven districts across seven states as described in the next section.

### Programme bottlenecks in training, supervision, and supplies

A systematic assessment of the programme implementation was carried out among seven early-implementing districts across seven states. A team of external supervisors visited the districts and collected information using semi-structured sets of questionnaires as follows: (a) Interviews with district health and nutrition officials; (b) Interviews with faculty and review of records of the District Training Centre; (c) Interviews with PHC staff and review of records of one PHC from each administrative block; (d) Interviews with ANMs and review of records of two sub-centres from each block; (e) Interviews with AWWs and review of records of two Anganwadis from each sub-centre area; and (f) Home-visits and observation of worker-family interface of 1-2 families with newborns.

Each component (training, supervision, supplies, and performance) had a set of five indicators, i.e. 20 indicators for four components. A score of 0-2 was assigned against each indicator (0=no implementation; 1=inadequate implementation; and 2=fair implementation). Each member of the team reviewed the collected information. The team jointly assigned the final score by consensus. Finally, each component received a score that was the total of all the scores of its indicators: a possible score from0-10. For depiction purposes, each indicator was given a colour code: green=2, yellow=1, and red=0. Similarly, each component was also given a colour code: red=0-5 (poor), yellow=6-8 (average), and green=9-10 (good).

**Table 1.** Summary of methodology

Information	Indicators	Source of data/methodology
Coverage and pace of training	Number of districts implementing IMNCI* Number of districts in different phases of implementation Numbers (%) of workers trained, by category of workers Average number of workers trained per year per district	Analysis of programmatic data on IMNCI progress from all implementing districts (from reports of supervisors and programme managers)
Quality of training	Median quality score on training programmes	Data from quality checks on a random subset of about 70 training programmes across 14 states, using a standard tool
Performance of trained health workers	% of births where newborns visited within 24 hours* % of births where newborns visited 3 times within 10 days* % of workers appropriately classified, identified treatment, and counselled	Data from supervisors and data from programme reports (coverage of home-visits for newborns) Follow-up after training by supervisor: visits to 3 states (quality of care to sick children)
Programme bottlenecks in the following domains: Training, Supervision, Supplies, and Performance	Scores on each of these domains	Rapid qualitative programme assessment of IMNCI in 7 districts across 7 states
Effectiveness of IMNCI implementation in changing key newborn and childcare practices and services	% of deliveries conducted in a health facility (institutional delivery)* % children initiated breastfeeding within 1 hour (early initiation of breastfeeding)* % of children exclusively breastfed till 6 months (exclusive breastfeeding)* % of children, aged 12-23 months, fully immunized (full immunization)* % of children with diarrhoea in the past two weeks who received ORS (ORS-use rates)* % of children with ARI in the past two weeks who sought care*	Change in coverage of key indicators in 12 early-implementation IMNCI districts between DLHS II (2003-2004) and DLHS III (2007-2008) compared to the change in same indictors in 12 control districts

*Those indicators that were included in the RCH II programme for assessing the effectiveness of the programme;

ARI=Acute respiratory infection;

DLHS=District Level Houshold Survey;

IMNCI=Integrated Management of Neonatal and Childhood Illness;

ORS=Oral rehydration salt solution;

RCH=Reproductive and child health

### Effectiveness in improving coverage ofnewborn and childcare practices

The 12 districts that had initiated IMNCI in 2005 were selected (subsequently referred to as ‘early’ IMNCI districts). Data from two rounds of the District Level Household Surveys (DLHS) were used for the analysis. For each ‘early’ IMNCI district, a control district from the same state, matched on IMR and proportion of the scheduled caste or scheduled tribe, was identified. ‘Early’ IMNCI districts and control districts were compared for changes in the coverage levels of key child-health indicators during 2005-2007. Weighted averages of the percentage change in coverage levels were calculated for the intervention and control districts. The net difference in changes in coverage was then compared between the intervention and the control districts using linear regression adjusting for clustering and for sampling weights. The key indicators analyzed to assess the effectiveness of the programme are listed in Table 1, which also summarizes the methodology of the assessment.

## RESULTS

### Coverage, pace, and quality of training

By June 2009, the IMNCI programme was being implemented in about 223 (35.56%) of the 627 districts of the country. Of these districts, 39 were in the introduction phase, 111 in the early-implementation phase, 30 in the expansion phase, and 43 in the consolidation phase. By this date, 202,015 workers had been trained. Seventy percent of the workers trained under the programme were CHWs: AWWs and ASHAs (Fig.).

The number of workers trained per year per district ranged from 208 to 1,285 across different states, with a median of 562. Only two states, i.e. Chattisgarh and Gujarat, were able to train more than 1,000 workers per year per district (Table 2).

**Fig.**
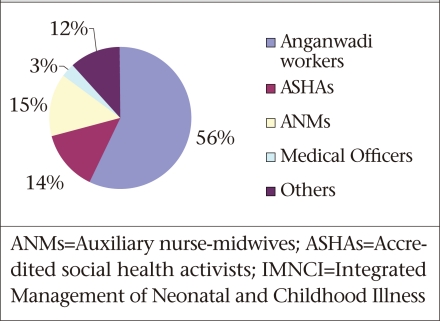
Distribution of category of workers trained in IMNCI, 2004-2008 (total 202,015 workers in 223 districts)

Based on assessment of the quality of training from 70 training programmes for frontline workers across 14 states, the median quality score was 88 (out of 100).

### Performance of trained health workers and community health workers

#### Coverage of home-visits

About 65% of all expected births were captured in the reporting period [interquartile range (IQR): 38.7-86.6%]. These amounted to about 1.1 million newborns across these 99 districts in the reporting period. Of these births, 72,257 (65.5%) were visited at home within 24 hours of birth, and 696,066 (63.1%) were visited three times within 10 days of birth. About 18% of all the newborns were referred. During the same period, the frontline workers also assessed 790,242 older children and advised referral to about 110,140 (13.9%) of them (Table 3A).

#### Quality of home-visits

Of 1,591 health workers who observed across three districts in three states, 1,302 (81.8%) appropriately classified the children, and 1,271 (80.3%) identified appropriate treatment (Table 3B).

Of the seven district where a systematic programme review was conducted, three fared good on the performance of trained workers; three fared average, and two fared poor. Despite the variations in the performance of workers across the districts, there was a pattern. While weighing and assessment were fair, handwashing and counselling were weak components. Supervision was the weakest component of programme implementation across all the districts (Table 4).

### Programme bottlenecks in training, supervision, and supplies

Table 4 summarizes the bottlenecks in programme implementation, as identified by programme reviews in seven districts. Training was assessed to be good in six of seven districts and average in the remaining district. However, supervision was assessed to be poor in most (6/7) districts. Supplies were assessed as good in five of the seven districts and poor in the remaining two districts.

### Effectiveness in improving coverage ofnewborn and childcare practices

Table 5 summarizes the comparison between changes in the coverage of key indicators between the IMNCI districts and the control districts. In the intervention districts, the coverage levels of all the indicators were higher at the endline compared to the baseline; the differences were least in the case of ORS-use rates and immunization (1.6% and 3.8% respectively). During this period, there was also an increase in the coverage levels of all the indicators in the control districts, except for the proportion of children with acute respiratory infection (ARI), who sought care. Except for the indicator—percentage of children fully immunized—the net change in the coverage levels of all other indicators was higher among the intervention districts than among the control districts. After adjusting for clustering and sampling weights, however, the difference was significant only for the proportion of children with ARI seeking care (net difference: 17.8%; 95% CI 2.3-33.2, p=0.026).

**Table 2.** Coverage and pace of IMNCI training

A. Coverage of IMNCI training by state (14 selected states)			
State	No. of IMNCIdistricts (n=203)	% of workers trained (of all health workers in district)	Average no. of workers trained per year per district
Andhra Pradesh	2	33	1,062
Assam	9	41	247
Bihar	15	22	440
Chattisgarh	18	90	1,285
Gujarat	18	75.4	1,043
Jharkhand	13	20	490
Karnataka	14	25	328
Madhya Pradesh	15	64.3	528
Maharashtra	25	35	547
Orissa	16	42.6	584
Rajasthan	17	49.4	208
Tamil Nadu	20	51.5	656
Uttar Pradesh	18	50.9	590
West Bengal	3	62.5	372
B. Pace of training by year of initiation			
Year of initiation	No. of districts (n=198)*	% of workers trained (by September 2009)	No. of workers trained per month
2005	12	82.9	949
2006	20	88.7	1,230
2007	78	62.5	4,700
2008	88	19.4	2,885

*Excludes districts that initiated IMNCI in 2009;

IMNCI=Integrated Management of Neonatal and Childhood Illness

## DISCUSSION

A large number of workers have been trained on IMNCI in India. Compared to the target of implementing IMNCI in 125 districts by 2010, the strategy was introduced in 223 districts, of which 73 (32.73%) districts had trained more than 50% of their total health workforce. The study suggests that the training programme led to the uptake of skills among the trained workers and, importantly, also to contacts of the newborns with the trained health workers within the first week of their life. The comparison of the DLHS data also provides an early evidence of the effectiveness of the programme on the coverage of some key newborn and childcare practices, such as care-seeking for ARI, institutional delivery, early initiation of breastfeeding, and exclusive breastfeeding. When compared with the improvements in the control districts during the same period, improvements in the intervention districts were higher on all indicators, except the immunization coverage, although improvement in care-seeking for ARI was the only indicator that assumed statistical significance. No apparent explanation could be found for the poor performance of the intervention districts on the immunization coverage.

**Table 3.** Performance of health workers after training (coverage and quality)

A. Coverage of home-visits						
Implementation status (based on reports from 99 districts)						No. (%)
No. of livebirths reported						1,102,573
Median % of expected births reported (IQR)						65.3 (38.7-86.6)
No. (%) of births where newborn were visited within 24 hours						722,571 (65.5)
No. (%) of births where newborn were visited 3 times within 10 days						696,060 (63.1)
No. (%) of newborns referred to a health facility						85,536 (18.0)
B. Quality of care for sick children in 3 states						
State	Appropriate classification		Appropriate treatment identification		Appropriatecounselling	
	No.	%	No.	%	No.	%
Rajasthan (n=178)	128	71.9	129	72.5	134	75.3
Bihar (n=989)	811	82	753	76.1	NA	
Orissa (n=424)	363	88.3	395	93.2	NA	

IQR=Interquartile range;

NA=Not available

**Table 4.**
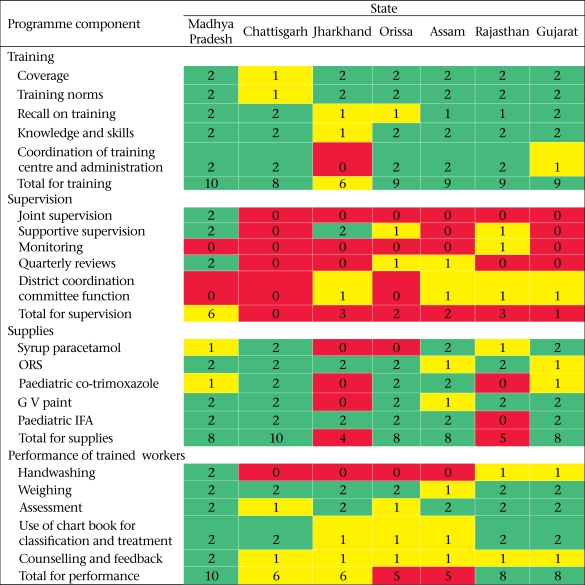
Rapid assessment of IMNCI implementation by external reviewers in seven selected IMNCI districts of India, 2009*

*Green denotes good; yellow denotes average; and red denotes poor;

IFA=Iron-folic acid;

IMNCI=Integrated Management of Neonatal and Childhood Illness;

ORS=Oral rehydration salt solution

There are several areas of concern. While a large number of workers have been trained, at the current pace, considering that the average number of workers to be trained in each district is close to 2,000, it would take about 3-4 years to complete the training load. Some states have been able to scale up training programmes at a faster pace using different approaches, such as engagement of private sectors (Gujarat) and full-time stand-alone trainers (Mayurbhanj, Orissa). There is a need to explore these approaches and adapt these in different states and districts.

**Table 5.** Change in coverage of key indicators in early-implementation IMNCI districts compared to control districts, 2004-2008

Indicator	Intervention districts (n=12)	Control districts (n=12)	Net difference in change (%) between intervention and control districts	95% CI**
	Change (%) between DLHS II and DLHS III*	Change (%) between DLHS II and DLHS III*		
% of institutional deliveries	9.2	5.0	4.2	-3.8,12.2
% of children for whom breastfeeding was initiated within 1 hour	18.1	13.6	4.4	-6.9,15.8
% of children exclusively breastfed	30.0	24.3	5.8	-6.3,17.9
% of children fully immunized	3.8	11.1	-7.3	-25.2,10.6
% of children with diarrhoea who received ORS	1.6	0.7	0.9	-10.0,11.7
% of children with ARI sought care	6.7	-11.1	17.8	2.3,33.2***

*Weighted average;

**Using linear regression adjusted for cluster and sampling weights;

***p=0.026;

ARI=Acute respiratory infection;

CI=Confidence interval;

DLHS=District Level Household Survey;

IMNCI=Integrated Management of Neonatal and Childhood Illness;

ORS=Oral rehydration salt solution

The information on the coverage of home-visits is based on those districts from where information was available. Little information was available on the coverage from other districts. Rapid programme assessment also highlighted that reporting on implementation has been a weak component of the programme and requires mainstreaming with the national HMIS.

Even in the reporting districts, home-visits reached only 64% of births. The newborns not reached are likely to be the ones who are most vulnerable. There are several reasons why home-visits did not reach about one-third of all newborns: absence of workers in several villages, poor supervision, and lack of motivation of the workers for this additional task. In some states, such as in Gujarat, the frontline workers are being given incentives for home-visits. While assessment of the impact of incentives on the performance of the frontline health workers is beyond the scope of this study, experiences from similar settings suggest that monetary and non-monetary incentives help improve the performance of CHWs (13).

Poor supervision and monitoring and poor availability of logistics and supplies were the major bottlenecks in programme implementation. The multi-country evaluation of IMCI had strikingly similar findings. In each of the countries evaluated, improvement in the performance of health workers was found to be ‘strong’. However, the health-system strengthening component of the programme was poorly implemented (14). It was recommended that public accountability should be enhanced to identify and address the bottlenecks of delivery (15). A recent analysis of the effectiveness of the Accelerated Child Survival and Development Programme in Africa also highlighted the role of systemic issues affecting the effectiveness of the child-survival programmes (16).

In Tanzania, a decentralized approach was adopted to improve supervision to overcome the barriers of distance, transportation, and cost. Results of an initial evaluation of IMCI implementation in Tanzania showed significantly improved supervision in implementing districts than in control districts. However, it was reported subsequently that even integrated (and decentralized) approaches to supervision in Tanzania and Uganda proved too ambitious, particularly with respect to the need to include clinical observations and feedback as part of all supervisory visits (14,17,18). Since the IMNCIprogramme in India is largely dependent on frontline workers, supervision of a large number of workers scattered across a large number of villages is even more challenging. Some districts have tried innovative measures to strengthen the supervision of the IMNCI-trained workers (Box). Such innovative methods need to be quickly reviewed and scaled up to ensure the appropriate implementation of the programme. There are also potential ways to improve the supplies and logistics for the IMNCI-trained workers, such as stocking supplies with Village Health and Sanitation Committees, as has been tried in an ongoing randomized controlled trial in India that aims to assess the effectiveness of IMNCI on newborn and child mortality (Bhandari N. Personal communication, 2011).

**Box. Innovative ways of supportive supervision**

**Peer supervision:** One of the trained frontline workers is engaged and trained as supervisor to support her peers, with some allowance for mobility

**Using trainers as supervisors:** The trainers train for half a month and supervise the trained workers in the remaining half

Almost 18% of the newborns were reported to have been referred. The present study, however, could not ascertain the compliance of the families with the referral. The referral rate appeared to be high and may lead to over-referral to the facilities. If not found sick enough for admission, this might lead to loss of credibility of the community-based workers. One of the reasons for the high rate of referrals could be high sensitivity and low specificity of the criteria used by the IMNCI approach for referral. A recent study has shown that, with fewer signs, specificity of the referral criteria for newborns can be enhanced while retaining the sensitivity (19).

### Limitations

There are certain limitations of the study. The study was conducted based on information from multiple sources, at different times and, thus, cannot be equated with a structured programme evaluation. While some information (e.g. on training) was available for all the districts, information on implementation was available on a subset. Information onimplementation was, for example, available only from 99 of the 223 districts. Implementation in the reporting districts is likely to be better than from the ‘non-reporting’ districts. Despite these limitations, the study provides evidence and insights for improving the programme performance.

### Conclusions

India has made significant investments in terms of time, effort, and money to roll out the IMNCI programme in the country. While the roll-out is slow, the detailed operational plans ensured the reasonable quality of the training programmes and have enhanced the skills of the workers. The contacts between the trained workers and the newborns and their families have also increased. However, to ensure that the investments made so far lead to reduction in newborn and infant mortality in the medium to long term, some urgent actions are required.

First, the programme assessment, as conducted in the selected districts, needs to be conducted in all the states and districts to identify the programme bottlenecks. Such an assessment should lead to corrective actions. Second, incentives to the frontline workers for home-visits to all newborns should be strongly considered for increasing the coverage of home-visits. Third, for ensuring the regular availability of key supplies, the Village Health and Sanitation Committees should be entrusted to stock supplies for the frontline workers. Fourth, besides strengthening the line supervisory structures, engagement of non-governmental organizations, universities, and other institutions should be considered in a systematic manner to ensure supportive supervision to the trained workers, at least for an initial few years after the training. Regular reviews of the programme at the national, state and district levels would further enhance its importance and also identify the critical bottlenecks in its implementation in a timely manner. Last, but most importantly, being intensive in nature focusing on a very large number of CHWs, the programme would require significant time to start making an impact on infant mortality rates. Ensuring quality implementation of such a time-and effort-intensive programme would, therefore, require patience and persistence among the policy-makers and programme managers at all levels.
